# The Microbiome of Neotropical Water Striders and Its Potential Role in Codiversification

**DOI:** 10.3390/insects11090578

**Published:** 2020-08-31

**Authors:** Anakena M. Castillo, Kristin Saltonstall, Carlos F. Arias, Karina A. Chavarria, Luis A. Ramírez-Camejo, Luis C. Mejía, Luis F. De León

**Affiliations:** 1 Centro de Biodiversidad y Descubrimiento de Drogas, Instituto de Investigaciones Científicas y Servicios de Alta Tecnología (INDICASAT-AIP), P.O. Box 0843-01103 Panamá 5, Panama; anakenamar@gmail.com (A.M.C.); ramirezcamejo@gmail.com (L.A.R.-C.); lmejia@indicasat.org.pa (L.C.M.); 2 Department of Biotechnology, Acharya Nagarjuna University, Guntur 522 510, Andhra Pradesh, India; 3 Smithsonian Tropical Research Institute, P.O. Box 0843-03092 Amador, Naos, Panama; SaltonstallK@si.edu (K.S.); AriasC@si.edu (C.F.A.); kari07cha@berkeley.edu (K.A.C.); 4 Department of Biology, University of Massachusetts Boston, Boston, MA 02125, USA; 5 Department of Civil and Environmental Engineering, University of California, Berkeley, CA 94720, USA; 6 Coiba Scientific Station (COIBA-AIP), City of Knowledge, P.O. Box 0843-01853 Balboa, Panama

**Keywords:** amplicon sequence variants (ASVs), bacterial community, microbiome, Neotropical, codiversification, water striders, *Wolbachia*

## Abstract

**Simple Summary:**

Insects host a highly diverse bacterial community. Although we have a good understanding of the role that this microbiome plays in insects, the composition and diversity of microbiomes associated with Neotropical freshwater insects is virtually unknown. Here, we describe, for the first time, the microbiome associated with six species of Neotropical water striders in Panama. We also performed phylogenetic analyses to explore potential codiversification or coevolution between water strider species and their associated microbiome. We found a diverse microbiome associated with the six species of water striders, with the dominant bacterial taxa belonging to the phyla Proteobacteria and Tenericutes. Although some bacterial lineages were shared across species, some lineages were also uniquely associated with different water strider species. Our results suggest that both environmental variation and host phylogenetic identity are important drivers of the microbiome associated with water striders. Understanding the evolution of the host-microbiome interaction is crucial to our understanding of Neotropical freshwater ecosystems.

**Abstract:**

Insects host a highly diverse microbiome, which plays a crucial role in insect life. However, the composition and diversity of microbiomes associated with Neotropical freshwater insects is virtually unknown. In addition, the extent to which diversification of this microbiome is associated with host phylogenetic divergence remains to be determined. Here, we present the first comprehensive analysis of bacterial communities associated with six closely related species of Neotropical water striders in Panama. We used comparative phylogenetic analyses to assess associations between dominant bacterial linages and phylogenetic divergence among species of water striders. We found a total of 806 16S rRNA amplicon sequence variants (ASVs), with dominant bacterial taxa belonging to the phyla Proteobacteria (76.87%) and Tenericutes (19.51%). Members of the α- (e.g., *Wolbachia*) and γ- (e.g., *Acinetobacter*, *Serratia*) Proteobacteria, and Mollicutes (e.g., *Spiroplasma*) were predominantly shared across species, suggesting the presence of a core microbiome in water striders. However, some bacterial lineages (e.g., *Fructobacillus*, *Fluviicola* and *Chryseobacterium*) were uniquely associated with different water strider species, likely representing a distinctive feature of each species’ microbiome. These findings indicate that both host identity and environmental context are important drivers of microbiome diversity in water striders. In addition, they suggest that diversification of the microbiome is associated with diversification in water striders. Although more research is needed to establish the evolutionary consequences of host-microbiome interaction in water striders, our findings support recent work highlighting the role of bacterial community host-microbiome codiversification.

## 1. Introduction

Insects host a highly diverse microbiome, which plays a crucial role in insect life. This bacterial community is involved in a variety of functions, ranging from food processing [1,2], to protection against pathogens [2,3,4,5,6] and regulation of developmental and life cycles [2,7]. In addition, recent studies have highlighted the contribution of the microbiome to diversification [8,9,10], including the evolution of reproductive isolation between species [10,11,12]. For instance, the presence of bacterial taxa such as *Wolbachia*, *Rickettsia* and *Cardinium* have been associated with mating incompatibility [13,14,15,16,17] and speciation in many insects (e.g., the plant-sap feeding Hemiptera and gall wasp (Cynipidae) [12,18,19,20]. Thus, understanding the nature and consequences of host-microbiome interactions in insects is crucial to our understanding of diversification in nature. However, despite the functional and evolutionary consequences of host-microbiome interactions [9,16,21], the composition and diversity of the bacterial community associated with Neotropical freshwater insects remains unexplored. In addition, questions regarding the evolution of host-microbiome interaction and its potential association with diversification in Neotropical freshwater insects have received little attention to date. Here, we advance these issues by assessing bacterial community composition and diversity associated with six closely related species of Neotropical water striders in Panama. We also use a comparative phylogenetic approach to test for associations between dominant bacterial linages and genetic divergence among species of water striders.

Water striders (family Gerridae) are a conspicuous group of semi-aquatic insects that are typical of freshwater and estuarine ecosystems. They are found in a variety of environments including rivers, streams, lakes and even the open ocean [22,23]. A prominent feature of water striders is their ability to walk on water via specialized hydrophobic legs that distribute their weight over a large surface area and take advantage of the high surface tension of water [22,23]. Water striders are dominant predators, providing a crucial functional role to aquatic ecosystems [23,24,25,26,27]. Taxonomically, they are highly diverse, with nearly 450 known species [23], and many more remaining to be described, particularly in the Neotropics. We have taken advantage of this diversity and specialized life strategy to explore host-microbiome associations in Neotropical water striders.

## 2. Materials and Methods

### 2.1. Sampling Sites

**Figure 1:**
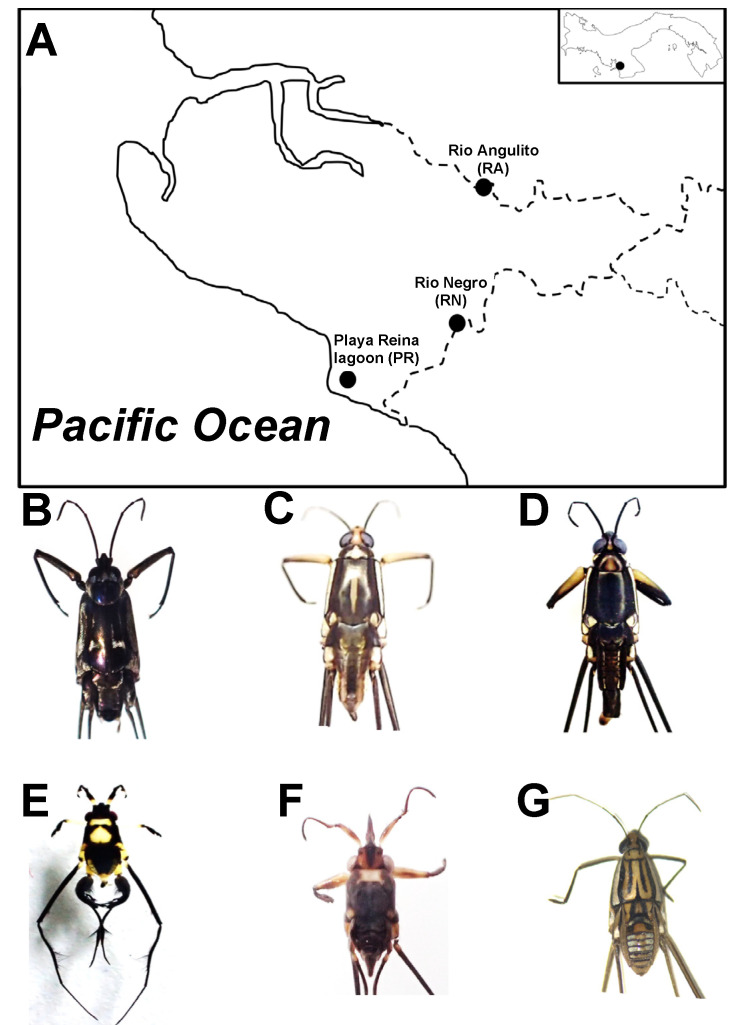
Sampling sites (**A**) and water striders species *Platygerris assimetricus* (Hungerford, 1932; **B**), *Potamobates horvathi* (Esaki, 1926; **C**), *Potamobates tridentatus* (Esaki, 1926, **D**), *Rheumatobates bergrothi* (Meinert, 1895; **E**), *Rheumatobates ornatus* (Polhemus and Cheng, 1976; **F**), *Telmatometra withei* (Bergroth, 1908; **G**). Photo credits: Pamela Polanco and Anakena Castillo.

We collected water striders from three sites located in Llano de Catival on the Western Azuero Peninsula on the Pacific coast of Panama (Figure 1A). The three sites are relatively close to each other (4 to 5 km), but vary in salinity levels due to sea water intrusion. Playa Reina lagoon (PR; 7°37′31.1″ N, 81°00′16.7″ O) has salinity levels ranging from 0.4 to 11 ppt, sandy substrate, and is surrounded by a mix of mangrove (*Rizhophora mangle*) and cativo (*Prioria copaifera*) forest. Río Angulito (RA; 7°38′22.0″ N, 80°58′17.0″ O) represents a typical estuarine site with salinity levels ranging from 0.1 to values >10 ppt. This site has a combination of rocky and sandy substrate, and is surrounded by mangrove and cativo trees and secondary forest. Río Negro (RN; 7°38′22.0″ N, 80°58′36.6″ O) is a typical freshwater river, with salinity levels ranging from 0 to 0.4 ppt. This site has gravel substrate and is surrounded by secondary forest.

### 2.2. Water Strider Species

We visited each site in December 2018 or January 2019 and collected a minimum of three adult individuals of six species of water striders: *Platygerris assimetricus* (RN), *Potamobates horvathi* (RA, RN), *Potamobates tridentatus* (RA), *Rheumatobates bergrothi* (PR), *Rheumatobates ornatus* (PR) and *Telmatometra withei* (PR, RA, RN) (Figure 1B–G; Table 1). Of the six water strider species sampled across sites, only three species were present at any given site, and only one species (*T. withei*) was present at all three sites. This pattern of species assemblage is likely due to habitat preference [22,23,28], given that our sampling sites included both fresh and brackish water sites (Figure 1A).

### 2.3. DNA Extraction and Amplification

Before DNA extraction, we surface-sterilized each insect by submerging it in 70% ethanol for 1 min, then rinsing three times in sterile water [29]. Whole individuals were then immersed in 0.01 M solution of sterile phosphate buffered saline (PBS) at 1× for 5 min [29] and macerated with a pestle in a 1.5 mL tube. DNA was extracted using a DNeasy Blood & Tissue Kit (Qiagen, Valencia, CA, USA), following the manufacturer’s protocol, with a final elution volume of 100 μL in buffer AE (buffer AE (elution buffer for genomic DNA).

To characterize the bacterial community associated with water striders, we used 16S rDNA primers (515 F and 806 R) [30] to amplify a 251 bp portion of the V4 region, which is one of the most effective regions for assessing bacterial diversity [31]. Triplicate PCR amplifications were prepared in 11 μL reaction volumes, containing 4.0 μL of molecular water, 5 μL of Platinum 2× Mastermix (ThermoFisher, Foster City, CA, USA), 0.5 μL primers 515F and 806R which included a partial Illumina adapter on their 5′ ends, and 1 μL DNA extract. Reaction conditions included a denaturation step of 94 °C for 3 min, followed by 35 cycles of denaturation at 94 °C for 45 s, annealing at 50 °C for 1 min, and elongation at 72 °C for 1.5 min, followed by a 10 min final elongation at 72 °C. We ran 2 μL of the PCR products on an agarose gel to verify amplification.

### 2.4. Library Preparation

We pooled our PCR triplicates and performed a second PCR to add barcode indexes and Illumina adapters in 12 μL reactions using 4 μL of molecular grade water, 5 μL of Platinum^TM^ master mix (Thermo Fisher 2×), 0.5 μL of each index (Forward and Reverse) and 2 μL of pooled PCR product. PCR started with a denaturation step of 94 °C for 3 min, followed by 6 cycles of denaturation at 94 °C, for 45 s, annealing at 50 °C for 1 min and elongation at 72 °C for 1.5 min, and ending with a 10 min final elongation at 72 °C. All resulting PCR reactions were cleaned and normalized with PCR purification and normalization plates (Charm Biotech, San Diego, CA, USA). All samples were combined and the library was concentrated and clean using KAPA pure beads (Kapa BioSystems, Wilmington, MA, USA). Final library concentration was determined using a Qubit fluorometer (Turner BioSystems, Foster city, CA, USA) and quality was checked on a BioAnalyzer (Agilent). Finally, the library was sequenced on an Illumina MiSeq sequencing platform (Illumina Inc., San Diego, CA, USA), on a 2 × 250 bp pair end run.

### 2.5. Data Analysis

We used the Quantitative Insights Into Microbial Ecology (QIIME 2.0) pipeline to process all raw bacterial 16S rRNA sequences associated with water striders [32]. In brief, we used Divisive Amplicon Denoising Algorithm (DADA2) [33], as implemented in R package version 4.0.2 (Kongens Nytorv, Denmark) to dereplicate and quality filter sequences. Then, we imported the sequence table into QIIME2 for following analysis. Representative amplicon sequence variants (ASVs) were assigned taxonomic classification with the SILVA database [34,35]. All ASVs assigned to mitochondrial and chloroplast sequences as well as those with less than 10 counts were removed from the dataset. Finally, the all data files generated with Qiime 2 were uploaded to the R software [36], for further statistical analyses and plotting.

#### 2.5.1. Bacterial Diversity and Community Composition

Sequences were rarefied to a depth of 9000 sequence per sample before performing diversity estimates. To estimate alpha diversity based on ASVs, we calculated Faith’s phylogenetic diversity (Faith’s PD), followed by nonparametric Kruskal-Wallis to examine statistical differences among site and species. We then quantified beta diversity among sites and species within sites based on weighted UniFrac distance and visualized it using principle coordinates analyses (PCoA) as implemented in the ggfortify and ggplot2 package [37,38].

To quantify variation in bacteria community composition across sites and species, as well as among species within site, we performed ANOSIM analyses in the vegan package [39]. We ran 1000 permutations for each analysis. Given our low sample size (3–5 individual per species/site; Table 1), and the fact that not all water strider species were present at each sampling site, we were not able to include the species vs. site effect into a single variance analysis. To further explore the effect of site, we tested (using ANOSIM) for variation in the bacterial community associated with *T. withei,* the only species present at all three sampling sites. Finally, the number of shared and unique bacterial taxa across water strider species and sites were visualized with a Venn diagram using the VennDiagram package [40].

#### 2.5.2. Exploring Phylogenetic Associations between Water Striders and Associated Microbiome

To determine phylogenetic relationships among water strider species, we amplified the Cytochrome oxidase I (COI) region from each of the six species of water striders, using the primers set LCO1490/HCO2198 [41] and dgLCO1490/dgHCO2198 [42]. We followed similar PCR protocols as in De León et al., 2020 [43]. We aligned the sequences using MAFFT, and then built a phylogenetic tree following RA × ML bootstrapping with 1000 iterations in Geneious 10.0.6 [44], and following the model GTR GAMMA. Then, we mapped the relative abundance of ASVs of the 29 most common bacterial taxa onto the phylogeny of the six water strider species using the vegan [39] and gplots packages [45].

## 3. Results

After trimming and filtering we obtained a total of 738,729 bacterial sequences, with an average of 18,941 ± 937 per sample. A rarefaction curve at a sequencing depth of 9000 showed the majority of bacterial diversity associated with water striders was captured at a relatively low number of reads (Figure S1).

### 3.1. Community Composition and Diversity of Water Strider Microbiomes

**Figure 2:**
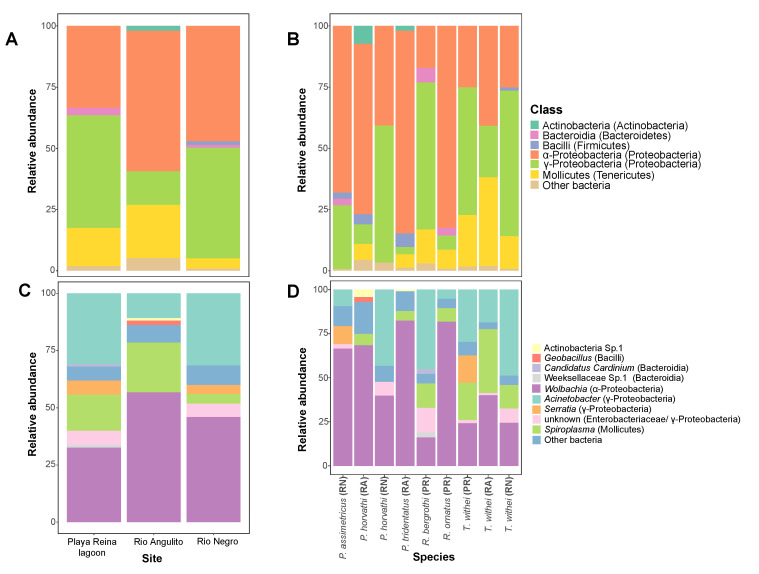
Relative abundance of dominant bacteria taxa associated with water striders. Abundance was estimated at the level of bacterial class across sites (**A**) and species within sites (**B**), as well as at the level of genus across sites (**C**) and species within sites (**D**). Only bacterial taxa with >5% sequence abundance are shown for both taxonomic levels.

After quality filtering, we found 806 ASVs associated with the six species of water striders. These ASVs were classified into 31 phyla, 59 classes, 138 orders, 222 families and 373 genera. Overall, the most abundant bacterial taxa were represented by the Phyla Proteobacteria (80.89%) and Tenericutes (13.81%), including the classes α- and γ-Proteobacteria and Mollicutes (Figure 2A,B). Other less abundant phyla such as Actinobacteria (Actinobacteria), Bacteroidetes (Bacteroidia) and Firmicutes (Bacilli) were also associated with some sites and water strider species (Figure 2A,B). Nine genera (Actinobacteria Sp. 1, *Geobacillus, Candidatus cardinium*, Weeksellaceae Sp. 1, *Wolbachia*, *Acinetobacter*, *Serratia,* Enterobacteriaceae (unknown) and *Spiroplasma*) were abundant across sites and species (Figure 2C,D).

**Figure 3:**
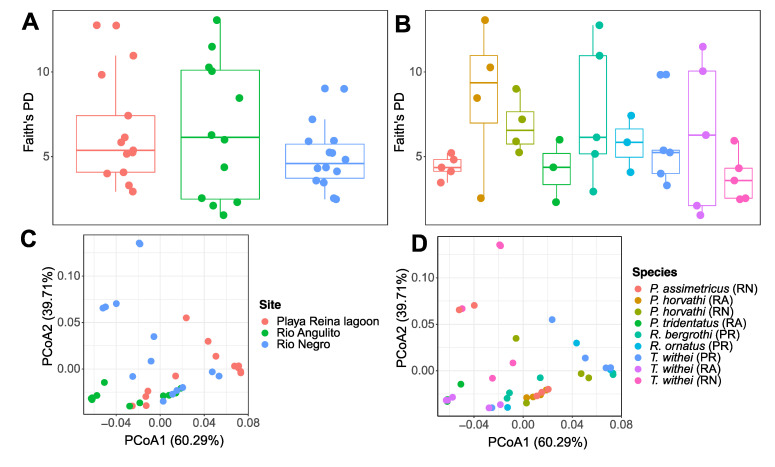
Bacterial diversity associated with water striders. Graphs represent estimates of alpha diversity based on Faith’s phylogenetic diversity (Faith’s PD) for each site (**A**) and species (**B**), as well as beta diversity principle coordinates analyses (PCoA) based on weighted UniFrac distance among sites (**C**) and species within sites (**D**).

Our estimates of alpha diversity did not show significant differences among sites or species (Kruskal-Wallis H, *p* > 0.05; Figure 3A,B). By contrast, beta diversity analyses based on weighted Unifrac distance showed significant differences among sites across species (ANOSIM statistic: R = 0.09, *p* < 0.05; Figure 3C), and among water strider species across sites (R = 0.36, *p* < 0.001; Figure 3D). However, in both cases the ANOSIM R statistic suggested that the microbial community share many taxa. Similarly, we found significant differences among species in Río Angulito and Negro (RA: R = 0.43, *p* < 0.05, and RN: R = 0.38, *p* < 0.01). By contrast, we did not find significant differences within Playa Reina lagoon (PR: R = 0.20, *p* > 0.05), and the bacterial communities associated with T. withei across the three sites (R = 0.02, *p* > 0.05), (Figure 3D).

**Figure 4:**
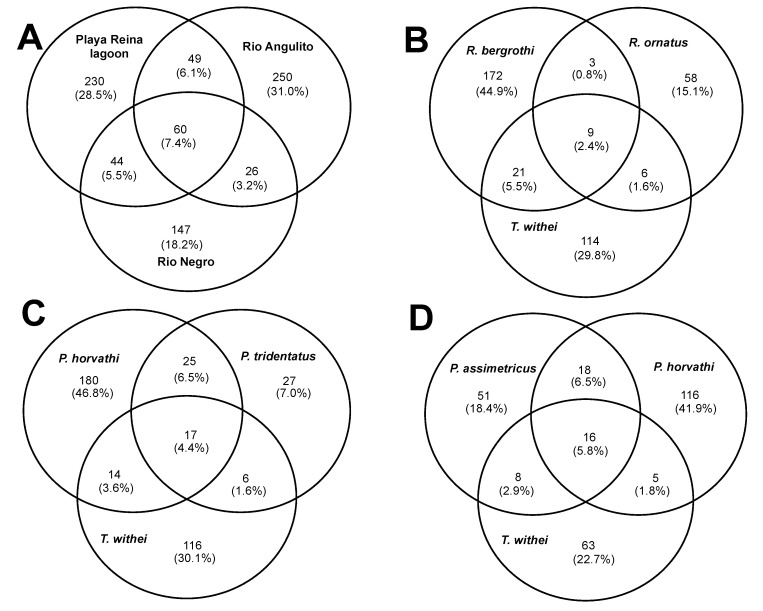
Distribution of bacteria amplicon sequence variants (ASVs) associated with water striders. Venn diagrams show the number (whole values) and proportion (in parenthesis) of unique and shared ASVs among sites (**A**) and species with sites, including Playa Reina lagoon (**B**), Río Angulito (**C**) and Río Negro (**D**).

The majority of ASVs (77.7%) were unique to a site and only 7.4% were shared among all sites, with both brackish water sites (Playa Reina lagoon and Río Angulito) showing the largest number of unique ASVs (Figure 4A). In addition, we found a large proportion of ASVs that were unique to each water strider species (Figure 4): 15.1–44.9% in Playa Reina lagoon (Figure 4B), 7.01–46.8% in Río Angulito (Figure 4C), and 18.4–41.9% in Río Negro (Figure 4D). Overall, however, few (2.4–5.8%) ASVs were shared among species within sites (Figure 4B,D).

### 3.2. Phylogenetic Associations

**Figure 5:**
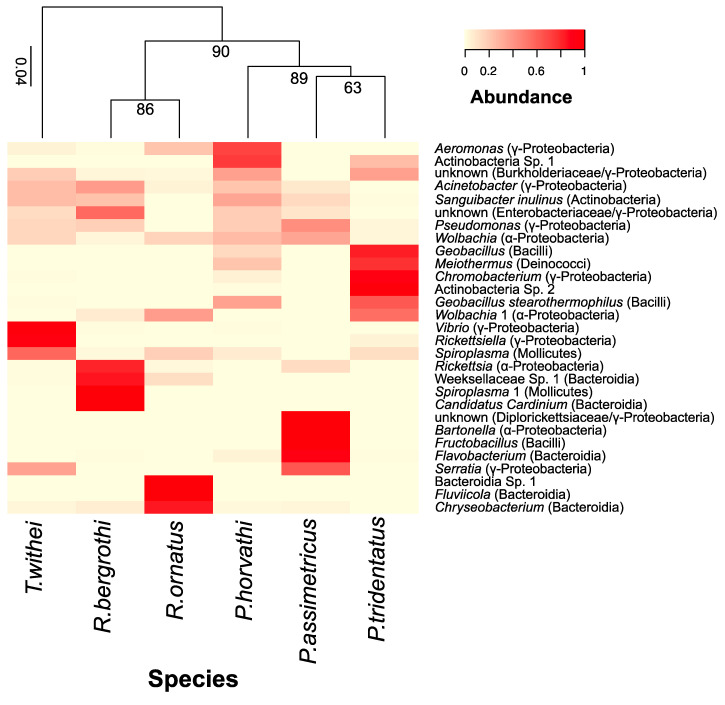
Amplicon sequence variants (ASVs) of dominant bacterial taxa associated with the phylogeny water striders. Only bacteria with a relative abundance of >0.5% were included in the analysis.

Our results showed that some bacterial taxa were uniquely associated with different water strider species (sequence found in at least 40% of the samples for given species). For instance, *P. assimetricus* showed bacterial taxa such as *Fructobacillus* (Figure 5). *P. tridentatus* showed unknown (Diplorickettsiaceae/Gammaproteobacteria) (Figure 5)*. R. ornatus* hosted *Fluviicola*, *Chryseobacterium* and Bacteroidia Sp. 1 (Figure 5). Finally, *T. withei* showed *Vibrio* and *Rickettsiella* (Figure 5). Overall, less than 24% of ASVs were shared among water strider species, and each water strider was often associated with a different bacterial cluster (Figure 5).

## 4. Discussion

The unique ecological niche occupied by water striders represents a fascinating opportunity to explore the evolution of host-microbiome interactions in freshwater and estuarine environments. Here, we assess, for the first time, the bacterial community associated with six closely related species of Neotropical water striders in Panama. We also explore potential phylogenetic associations between these bacterial communities and water strider species.

### 4.1. Bacterial Diversity and Core Microbiome of Water Striders

Overall, we found 806 ASVs of bacterial lineages associated with the six species of water striders. The most common and abundant bacterial taxa included phyla such as Proteobacteria and Tenericutes. These phyla were dominated by facultative endosymbionts, nonsymbiont, and pathogenic bacteria such as α- (e.g., *Wolbachia*) and γ- (e.g., *Acinetobacter*, *Serratia* and Enterobacteriaceae) Proteobacteria and Mollicutes (e.g., *Spiroplasma*). Given their high frequency across sites and species, these bacterial taxa represent the core microbiome of Neotropical water striders. These taxa have also been previously associated with both aquatic and terrestrial insects [46,47,48,49,50,51], suggesting that they are a common component of the microbiome of insects in general, and play important functional roles in their insect hosts [3,6,46,52]. For example, a recent study found that the genus *Wolbachia* is widespread in aquatic Hemipteran, including Gerridae, from Southwest Cameroon [53]. In addition, the genera *Wolbachia* and *Spiroplasma* are known to influence host ecology and evolution [1,3,6,53], and could be involved in diversification in water striders (see below).

We also found bacterial taxa that have not been previously associated with water striders. These include *Fructobacillus*, which was associated with *P. assimetricus*. This bacterial genus has also been reported in terrestrial insects [54,55,56,57], with some species offering protection against pathogens in bees such as the American foulbrood [58]. *Rickettsiella*, which was found in *T*. *withei*, is known to reduce mortality and decreases fungal sporulation in insects [59], however the genus is also an important pathogen in arthropods [60]. *Vibrio*, which was also associated with *T*. *withei*, is considered a pathogen of aquatic organisms [61,62], and can cause high mortality and severe economic losses in marine fisheries [61]. *Chryseobacterium*, which was found in *R*. *ornatus,* has been reported in other insects [63], and some of species can be pathogenic to humans and other animals [64]. However, the role of these genera in water striders is currently unknown.

Our most striking result was the high proportion of bacterial ASVs that were uniquely associated with different water strider species, with only 2.4–5.8% of ASVs being shared among species at each site. Although the functional consequences of this microbiome disparity is currently unknown, this finding suggests that species identity is likely a major factor driving microbiome diversity in water striders. Unfortunately, our small sample size prevented us from determining if this pattern is consistent across species or if some bacterial taxa show a stronger contribution to microbiome diversity than others. Microbiome composition could also be influenced by habitat type [65,66,67,68,69,70], particularly because we sampled one fresh and two brackish-water sites. Indeed, we found significant differences in the number of ASVs across sites, with brackish-water sites showing the highest number of unique bacterial taxa. This is consistent with previous work showing that some bacteria taxa belonging to α, γ and β-Proteobacteria present affinities for different levels of salinity, and that saline environments often host a higher bacterial diversity than freshwater habitats [67]. However, we believe that common environmentally derived bacterial taxa were infrequent in our samples, in part because we sterilized the external body of our water strider specimens before DNA extraction (i.e., our sampling was focused on the internal body). Thus, differences in microbiome composition across sites are likely confounded by the strong species effect (i.e., different water strider species were present at different sites), but more data are needed to disentangle these effects statistically.

Another factor influencing microbiome diversity is host diet [71], but we currently know little about the diet of our water strider species. However, given that water striders are opportunistic predators that feed mostly on insects that fall on the water surface [23], we may expect low variation in diet across species. Moreover, the fact that *T. withei*, the species that was present at all sites, showed low variation in microbiome composition across sites suggests that both diet and habitat type are less important in determining water strider microbiomes. However, further research is needed to confirm this possibility, particularly because some closely related species of aquatic Hemiptera (Veliidae and Gerridae) show differences in prey capture and feeding behavior [72].

### 4.2. Codiversification between Water Striders and Their Microbiome

Our phylogenetic analyses showed that some of the most abundant bacterial taxa were uniquely associated with different water strider species (Figure 5). In addition, it appears that some closely related species of water striders also host closely related bacterial microbiome (Figure 5). This was particularly evident for some bacterial taxa such as *Actinobacteria* (sp.1 and sp.2), *Geobacillus* and *Wolbachia*, which were hosted by *P. horvathi* and *P. tridentatus* (Figure 5). The results are consistent with recent studies showing strong associations between host phylogenetic divergence and phylogenetic divergence of the associated microbiome in several taxa, including humans [72,73], mice [74], birds [75], lizards [76] and insects [77].

Thus, codiversification between hosts and associated microbiomes appears to be a common evolutionary consequence of host-microbiome interaction. This is a tantalizing possibility in water striders, given that they host a high diversity of bacterial taxa that are known to affect several aspects of the reproductive biology in insects—including reproductive isolation. For example, we found a high abundance of *Wolbachia*, which has been associated with cytoplasmic incompatibility in other insect taxa [13,14,19,20].

Of particular interest are the genera *Wolbachia* and *Spiroplasma* which are common in insects [14,78,79], and are involved in a variety of functions [3,6]. This includes fecundity in some species of beetles (Family Curculionidae) [80,81], parthenogenesis [82], feminization [14,82,83], as well as cytoplasmic incompatibility [13,14]. *Wolbachia* has also been implicated in reproductive isolation and speciation in insects [78,84,85]. On the other hand, *Spiroplasma* is associated with male-killing [47,53,86]. Although more work is need to assess the evolutionary consequences of host-microbiome interaction in water striders, our results suggest the possibility that some bacterial taxa, such as *Wolbachia* and *Spiroplasma,* are involved in codiversification in water striders. Thus, future work should assess the diversity of these bacterial taxa in a larger number of water strider species. Experimental analyses are also necessary to confirm the potential role of these taxa in driving reproductive isolation.

## 5. Conclusions

In summary, our findings show that Neotropical water striders host a diverse bacterial community. Some of these bacterial taxa are uniquely associated with different water strider species, and these associations are likely influenced by both environmental context and host phylogenetic history. This suggests that diversification in water strider microbiomes is likely associated with host phylogenetic divergence. Assessing these associations is crucial to our understanding of the evolution host-microbiome interaction and its role in diversification and codiversification Neotropical freshwater organisms.

## Figures and Tables

**Table 1:** Number of individuals of six water strider species sampled at three sites on the Western Azuero Peninsula, Panama.

Site	Species	Number
Playa Reina lagoon	*Rheumatobates bergrothi*	5
	*Rheumatobates ornatus*	3
	*Telmatometra withei*	5
Río Angulito	*Potamobates horvathi*	4
	*Potamobates tridentatus*	3
	*Telmatometra withei*	5
Río Negro	*Platygerris assimetricus*	5
	*Potamobates horvathi*	4
	*Telmatometra withei*	5

## Supplementary Materials

The following are available online at https://www.mdpi.com/2075-4450/11/9/578/s1, Figure S1: Rarefaction curves of bacterial phylogenetic diversity (Faith’s PD, ±SE) associated with (**A**) our three sampling sites, and (**B**) six species of Neotropical water striders across the three sites. Raw sequence data and metadata are available at: https://doi.org/10.6084/m9.figshare.12855158.

## References

[1] Fukatsu T., Hosokawa T. (2002). Capsule-transmitted gut symbiotic bacterium of the Japanese common plataspid stinkbug, *Megacopta punctatissima*. Appl. Environ. Microbiol..

[2] Engel P., Moran N.A. (2013). The gut microbiota of insects—Diversity in structure and function. FEMS Microbiol. Rev..

[3] Hedges L.M., Brownlie J.C., O’Neill S.L., Johnson K.N. (2008). *Wolbachia* and Virus Protection in Insects. Science.

[4] Koch H., Schmid-Hempel P. (2011). Socially transmitted gut microbiota protect bumble bees against an intestinal parasite. Proc. Natl. Acad. Sci. USA.

[5] Motta E.V.S., Raymann K., Moran N.A. (2018). Glyphosate perturbs the gut microbiota of honey bees. Proc. Natl. Acad. Sci. USA.

[6] Teixeira L., Ferreira Á., Ashburner M. (2008). The bacterial symbiont *Wolbachia* induces resistance to RNA viral infections in *Drosophila melanogaster*. PLoS Biol..

[7] Coon K.L., Valzania L., McKinney D.A., Vogel K.J., Brown M.R., Strand M.R. (2017). Bacteria-mediated hypoxia functions as a signal for mosquito development. Proc. Natl. Acad. Sci. USA.

[8] Rennison D.J., Rudman S.M., Schluter D. (2019). Parallel changes in gut microbiome composition and function in parallel local adaptation and speciation. bioRxiv.

[9] Dillon R.J., Webster G., Weightman A.J., Dillon V.M., Blanford S., Charnley A.K. (2008). Composition of Acridid gut bacterial communities as revealed by 16S rRNA gene analysis. J. Invertebr. Pathol..

[10] Janson E.M., Stireman J.O., Singer M.S., Abbot P. (2008). Phytophagous insect-microbe mutualisms and adaptive evolutionary diversification. Evolution.

[11] Vavre F., Kremer N. (2014). Microbial impacts on insect evolutionary diversification: From patterns to mechanisms. Curr. Opin. Insect Sci..

[12] Schuler H., Egan S.P., Hood G.R., Busbee R.W., Driscoe A.L., Ott J.R. (2018). Diversity and distribution of *Wolbachia* in relation to geography, host plant affiliation and life cycle of a heterogonic gall wasp. BMC Evol. Biol..

[13] Poinsot D., Charlat S., Merçot H. (2003). On the mechanism of *Wolbachia*-induced cytoplasmic incompatibility: Confronting the models with the facts. BioEssays.

[14] Kikuchi Y. (2009). Endosymbiotic Bacteria in Insects: Their Diversity and Culturability. Microbes Environ..

[15] Perlman S.J., Hunter M.S., Zchori-Fein E. (2006). The emerging diversity of *Rickettsia*. Proc. R. Soc. B Biol. Sci..

[16] Moran N.A., McCutcheon J.P., Nakabachi A. (2008). Genomics and Evolution of Heritable Bacterial Symbionts. Annu. Rev. Genet..

[17] Zchori-Fein E., Perlman S.J. (2004). Distribution of the bacterial symbiont. Mol. Ecol..

[18] Poff K.E., Stever H., Reil J.B., Seabourn P., Ching A.J., Aoki S., Logan M., Michalski J.R., Santamaria J., Adams J.W. (2017). The native Hawaiian insect microbiome initiative: A critical perspective for Hawaiian insect evolution. Insects.

[19] Shropshire J.D., Bordenstein S.R. (2016). Speciation by symbiosis: The microbiome and behavior. MBio.

[20] Brucker R.M., Bordenstein S.R. (2012). Speciation by symbiosis. Trends Ecol. Evol..

[21] Lundgren J.G., Lehman R.M., Chee-sanford J. (2007). Bacterial Communities within Digestive Tracts of Ground Beetles (Coleoptera: Carabidae). Ann. Entomol. Soc. Am..

[22] Cheng L. (2005). Marine Insects.

[23] Stonedahl G.M., Lattin J.D. (1982). The Gerridae or Water Striders of Oregon and Washington (Hemiptera: Heteroptera). Tech. Bull. Agric. Exp. Station. Oregon State Univ..

[24] Izabella O., Pawel B., Piotr J., Lee S.D. (2007). Diet of Water Striders (*Gerris lacustris L*. 1758) in a Rice Field Near Seoul, Korea. J. Asia. Pac. Entomol..

[25] Merritt R.W., Cummins K.W., Berg M.B. (2008). An Introduction to Aquatic Insects of North America.

[26] Merritt R.W., Cummins K.W., Berg M.B., Novak J.A., Higgins M.J., Wessell K.J., Lessard J.L. (2002). Development and application of a macroinvertebrate functional-group approach in the bioassessment of remmant river oxbows in southwest Florida. J. N. Am. Benthol. Soc..

[27] Nummelin M. (1988). Waterstriders (Het: Gerridae) as predators of hatching mosquitoes. Bicovas.

[28] Pacheco B. (2012). Diversidad Taxonómica y Distribución de los Chinches Patinadores (Hemiptera: Gerridae) en Costa Rica. Licentiate Thesis.

[29] Chen B., Teh B.S., Sun C., Hu S., Lu X., Boland W., Shao Y. (2016). Biodiversity and Activity of the Gut Microbiota across the Life History of the Insect Herbivore *Spodoptera littoralis*. Sci. Rep..

[30] Caporaso J.G., Lauber C.L., Walters W.A., Berg-lyons D., Lozupone C.A., Turnbaugh P.J., Fierer N., Knight R. (2011). Global patterns of 16S rRNA diversity at a depth of millions of sequences per sample. Proc. Natl. Acad. Sci. USA.

[31] Mizrahi-Man O., Davenport E.R., Gilad Y. (2013). Taxonomic Classification of Bacterial 16S rRNA Genes Using Short Sequencing Reads: Evaluation of Effective Study Designs. PLoS ONE.

[32] Bokulich N.A., Kaehler B.D., Rideout J.R., Dillon M., Bolyen E., Knight R., Huttley G.A., Gregory Caporaso J. (2018). Optimizing taxonomic classification of marker-gene amplicon sequences with QIIME 2’s q2-feature-classifier plugin. Microbiome.

[33] Callahan B.J., McMurdie P.J., Rosen M.J., Han A.W., Johnson A.A., Holmes S.P. (2016). DADA2: High resolution sample inference from Illumina amplicon data. Nat. Methods.

[34] Suenami S., Konishi Nobu M., Miyazaki R. (2019). Community analysis of gut microbiota in hornets, the largest eusocial wasps, *Vespa mandarinia* and *V. simillima*. Sci. Rep..

[35] Nowinski B., Smith C.B., Thomas C.M., Esson K., Marin R., Preston C.M., Birch J.M., Scholin C.A., Huntemann M., Clum A. (2019). Microbial metagenomes and metatranscriptomes during a coastal phytoplankton bloom. Sci. Data.

[36] R Development Core (2008). R: A Language and Environment for Statistical Computing.

[37] Callahan B.J., Sankaran K., Fukuyama J.A., McMurdie P.J., Holmes S.P. (2016). Bioconductor workflow for microbiome data analysis: From raw reads to community analyses. F1000Research.

[38] Tang Y., Horikoshi M., Li W. (2016). Ggfortify: Unified interface to visualize statistical results of popular r packages. R J..

[39] Oksanen J., Blanchet F.G., Friendly M., Kindt R., Legendre P., Mcglinn D., Minchin P.R., O’hara R.B., Simpson G.L., Solymos P. (2017). Vegan: Community Ecology Package. https://cran.r-project.org/web/packages/vegan/index.html.

[40] Wilkinson L. (2012). Exact and approximate area-proportional circular Venn and Euler diagrams. IEEE Trans. Vis. Comput. Graph..

[41] Folmer O., Black M.B., Black M., Hoeh W., Lutz R. (1994). DNA primers for amplification of mitochondrial Cytochrome *c* Oxidase subunit I from diverse metazoan invertebrates. Mol. Mar. Biol. Biotechnol..

[42] Meyer C.P. (2003). Molecular systematics of cowries (Gastropoda: Cypraeidae) and diversification patterns in the tropics. Biol. J. Linn. Soc..

[43] De León L.F., Cornejo A., Gavilán R.G., Aguilar C. (2020). Hidden biodiversity in Neotropical streams: DNA barcoding uncovers high endemicity of freshwater macroinvertebrates at small spatial scales. PLoS ONE.

[44] Kearse M., Moir R., Wilson A., Stones-Havas S., Cheung M., Sturrock S., Buxton S., Cooper A., Markowitz S., Duran C. (2012). Geneious Basic: An integrated and extendable desktop software platform for the organization and analysis of sequence data. Bioinformatics.

[45] Warnes G.R., Bolker B., Bonebakker L., Gentleman R., Liaw W.H.A., Lumley T., Maechler M., Magnusson A., Moeller S., Schwartz M. (2016). Package “gplots”: Various R programming tools for plotting data. https://cran.r-project.org/package=gplots.

[46] Azambuja P., Feder D., Garcia E.S. (2004). Isolation of *Serratia marcescens* in the midgut of *Rhodnius prolixus*: Impact on the establishment of the parasite *Trypanosoma cruzi* in the vector. Exp. Parasitol..

[47] Montenegro H., Solferini V.N., Klaczko L.B., Hurst G.D.D. (2005). Male-killing *Spiroplasma* naturally infecting *Drosophila melanogaster*. Insect Mol. Biol..

[48] Minard G., Tran F.H., Raharimalala F.N., Hellard E., Ravelonandro P., Mavingui P., Valiente Moro C. (2013). Prevalence, genomic and metabolic profiles of *Acinetobacter* and *Asaia* associated with field-caught *Aedes albopictus* from Madagascar. FEMS Microbiol. Ecol..

[49] Sazama E.J., Bosch M.J., Shouldis C.S., Ouellette S.P., Wesner J.S. (2017). Incidence of *Wolbachia* in aquatic insects. Ecol. Evol..

[50] Kolasa M., Kubisz D., Mazur M.A., Ścibior R., Kajtoch Ł. (2018). *Wolbachia* prevalence and diversity in selected riverine predatory beetles (Bembidiini and Paederini). Bull. Insectol..

[51] González C.T., Saltonstall K., Fernández-marín H. (2019). Garden microbiomes of *Apterostigma dentigerum* and *Apterostigma pilosum* fungus-growing ants (Hymenoptera: Formicidae ). J. Microbiol..

[52] Abbott D.W., Boraston A.B. (2008). Structural Biology of Pectin Degradation by *Enterobacteriaceae*. Microbiol. Mol. Biol. Rev..

[53] Esemu S.N., Dong X., Kfusi A.J., Hartley C.S., Ndip R.N., Ndip L.M., Darby A.C., Post R.J., Makepeace B.L. (2019). Aquatic Hemiptera in Southwest Cameroon: Biodiversity of Potential Reservoirs of Mycobacterium ulcerans and multiple Wolbachia sequence types revealed by metagenomics. Diversity.

[54] Endo A., Tanizawa Y., Tanaka N., Maeno S., Kumar H., Shiwa Y., Okada S., Yoshikawa H., Dicks L., Nakagawa J. (2015). Comparative genomics of *Fructobacillus* spp. and *Leuconostoc* spp. reveals niche-specific evolution of *Fructobacillus* spp.. BMC Genom..

[55] Maddaloni M., Hoffman C., Pascual D.W. (2014). Paratransgenesis feasibility in the honeybee (*Apis mellifera*) using *Fructobacillus fructosus* commensal. J. Appl. Microbiol..

[56] Endo A., Maeno S., Tanizawa Y., Kneifel W., Arita M., Dicks L., Salminen S. (2018). Fructophilic lactic acid bacteria, a unique group of fructose-fermenting microbes. Appl. Environ. Microbiol..

[57] Endo A. (2012). Fructophilic lactic acid bacteria inhabit fructose-rich niches in nature. Microb. Ecol. Health Dis..

[58] Janashia I., Choiset Y., Rabesona H., Hwanhlem N., Bakuradze N., Chanishvili N., Haertlé T. (2016). Protection of honeybee *Apis mellifera* by its endogenous and exogenous lactic flora against bacterial infections. Ann. Agrar. Sci..

[59] Su Q., Zhou X., Zhang Y. (2013). Symbiont-mediated functions in insect hosts. Commun. Integr. Biol..

[60] Bouchon D., Cordaux R., Grève P., Zchori-Fein E., Bourtzis K. (2012). *Rickettsiella*, Intracellular Pathogens of Arthropods. Manipulative Tenants.

[61] Abdelaziz M., Ibrahem M.D., Ibrahim M.A., Abu-Elala N.M., Abdel-moneam D.A. (2017). Monitoring of different vibrio species affecting marine fishes in Lake Qarun and Gulf of Suez: Phenotypic and molecular characterization. Egypt. J. Aquat. Res..

[62] Hughes J., Lancaster J., Briers R.A. (2008). Population Genetic Structure in Stream Insects: What Have We Learned. Aquatic Insects: Challenges to Populations.

[63] Dugas J.E., Zurek L., Paster B.J., Keddie B.A., Leadbetter E.R. (2001). Isolation and characterization of a *Chryseobacterium* strain from the gut of the American cockroach, *Periplaneta americana*. Arch. Microbiol..

[64] Bloch K.C., Nadarajah R., Jacobs R. (1997). *Chryseobacterium meningosepticum*: An emerging pathogen among immunocompromised adults. Report of 6 cases and literature review. Medicine.

[65] Pruzzo C., Huq A., Colwell R.R., Donelli G. (2005). Pathogenic vibrio species in the marine and estuarine environment. Ocean. Health Pathog. Mar. Environ..

[66] Thompson F., Iida T., Swings J. (2004). Biodiversity of Vibrios. Microbiol. Mol. Biol. Rev..

[67] Morrissey E.M., Franklin R.B., Kent A. (2015). Evolutionary history influences the salinity preference of bacterial taxa in wetland soils. Front. Microbiol..

[68] Zhang M., Sun Y., Liu Y., Qiao F., Chen L., Liu W.T., Du Z., Li E. (2016). Response of gut microbiota to salinity change in two euryhaline aquatic animals with reverse salinity preference. Aquaculture.

[69] Gallardo K., Candia J.E., Remonsellez F., Escudero L.V., Demergasso C.S. (2016). The ecological coherence of temperature and salinity tolerance interaction and pigmentation in a non-marine *Vibrio* isolated from salar de atacama. Front. Microbiol..

[70] Truong A., Sondossi M., Clark J.B. (2017). Genetic characterization of *Wolbachia* from Great Salt Lake brine flies. Symbiosis.

[71] Krams I.A., Kecko S., Jõers P., Trakimas G., Elferts D., Krams R., Luoto S., Rantala M.J., Inashkina I., Gudrā D. (2017). Microbiome symbionts and diet diversity incur costs on the immune system of insect larvae. J. Exp. Biol..

[72] Ditrich T., Papáček M. (2016). Differences in prey capture in semiaquatic bugs (Heteroptera: Gerromorpha). Entomol. Sci..

[73] Falush D., Wirth T., Linz B., Pritchard J.K., Stephens M., Kidd M., Blaser M.J., Graham D.Y., Vacher S., Perez-perez G.I. (2003). Traces of Human Migrations in Helicobacter pylori Populations. Science.

[74] Moeller A.H., Gomes-Neto J.C., Mantz S., Kittana H., Segura Munoz R.R., Schmaltz R.J., Ramer-Tait A.E., Nachman M.W. (2019). Experimental Evidence for Adaptation to Species-Specific Gut Microbiota in House Mice. mSphere.

[75] Kropáčková L., Těšický M., Albrecht T., Kubovčiak J., Čížková D., Tomášek O., Martin J.F., Bobek L., Králová T., Procházka P. (2017). Codiversification of gastrointestinal microbiota and phylogeny in passerines is not explained by ecological divergence. Mol. Ecol..

[76] Ren T., Kahrl A.F., Wu M., Cox R.M. (2016). Does adaptive radiation of a host lineage promote ecological diversity of its bacterial communities? A test using gut microbiota of *Anolis* lizards. Mol. Ecol..

[77] Moran N.A., Sloan D.B. (2015). The Hologenome Concept: Helpful or Hollow?. PLoS Biol..

[78] Hilgenboecker K., Hammerstein P., Schlattmann P., Telschow A., Werren J.H. (2008). How many species are infected with *Wolbachia*?—A statistical analysis of current data. FEMS Microbiol. Lett..

[79] Gauthier J.P., Outreman Y., Mieuzet L., Simon J.C. (2015). Bacterial communities associated with host-adapted populations of pea aphids revealed by deep sequencing of 16S ribosomal DNA. PLoS ONE.

[80] Chen S.-J., Lu F., Cheng J.-A., Jiang M.-X., Way M.O. (2012). Identification and Biological Role of the Endosymbionts *Wolbachia* in Rice Water Weevil (Coleoptera: Curculionidae). Environ. Entomol..

[81] Zchori-Fein E., Borad C., Harari A.R. (2006). Oogenesis in the date stone beetle, *Coccotrypes dactyliperda*, depends on symbiotic bacteria. Physiol. Entomol..

[82] Stouthamer R., Breeuwer J.A.J., Hurst G.D.D. (1999). *Wolbachia Pipientis*: Microbial Manipulator of Arthropod Reproduction. Annu. Rev. Microbiol..

[83] Hiroki M., Kato Y., Kamito T., Miura K. (2002). Feminization of genetic males by a symbiotic bacterium in a butterfly, *Eurema hecabe* (Lepidoptera: Pieridae). Naturwissenschaften.

[84] Hurst G.D.D., Jigging F.M., Von Der Schulenburg J.H.G., Bertrand D., West S.A., Goriacheva I.I., Zakharov I.A., Werren J.H., Stouthamer R., Majerus M.E.N. (1999). Male-killing *Wolbachia* in two species of insect. Proc. R. Soc. B Biol. Sci..

[85] Telschow A., Hammerstein P., Werren J.H. (2005). The effect of *Wolbachia* versus genetic incompatibilities on reinforcement and speciation. Evolution.

[86] Harumoto T., Fukatsu T., Lemaitre B. (2018). Common and unique strategies of male killing evolved in two distinct *Drosophila* symbionts. Proc. R. Soc. B Biol. Sci..

