# Supplementary material for: The Microbiome of Neotropical Water Striders and Its Potential Role in Codiversification
Source: Insects. 2020 Aug 31;11(9):578. doi: 10.3390/insects11090578 (PMC7565411; doi:10.3390/insects11090578)
Supplement: Supplementary file 1 [file insects-11-00578-s001.pdf]

Figure S1

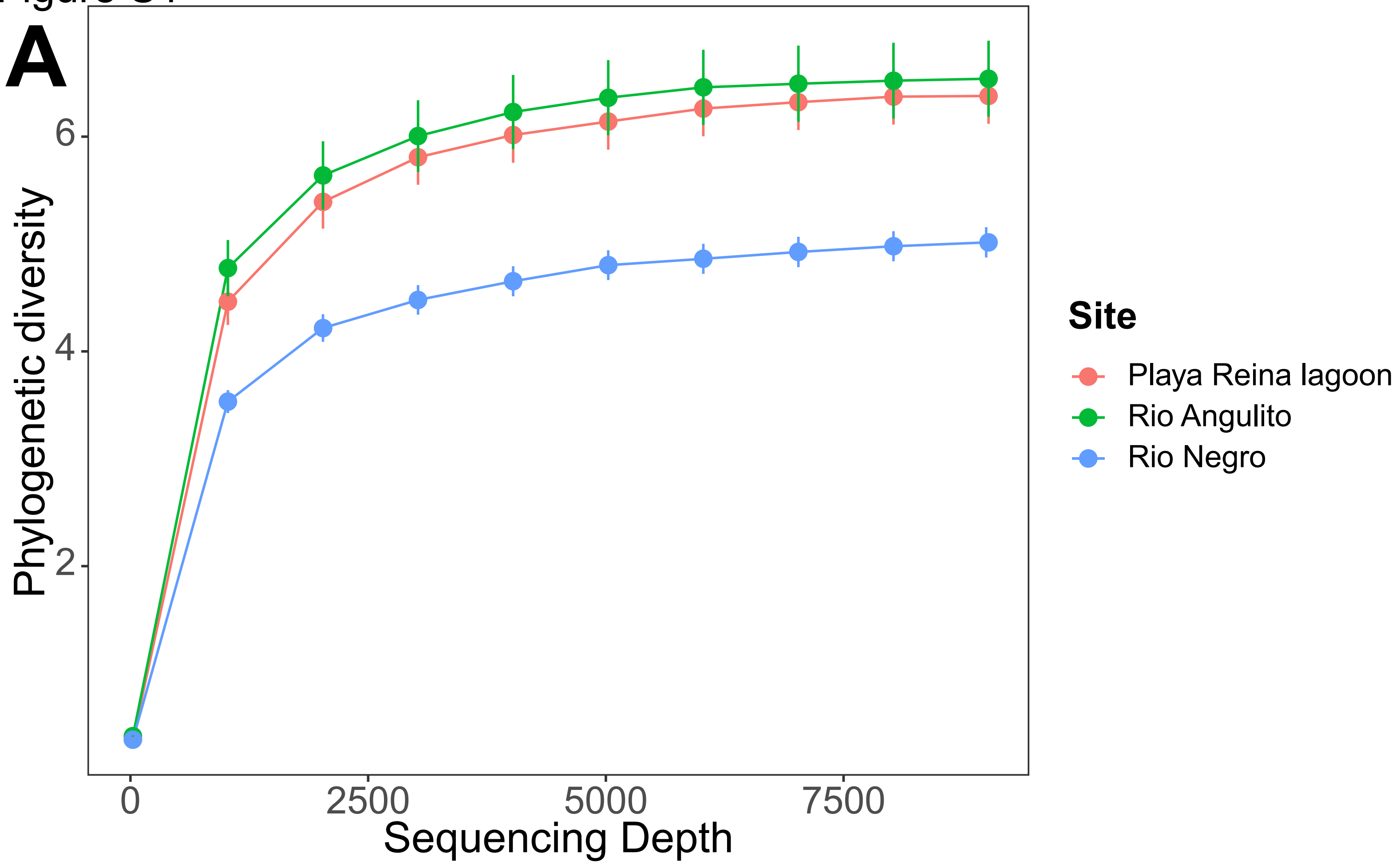

A
Phylogenetic diversity
6
4
2
0
2500
5000
7500
Sequencing Depth
Site
Playa Reina lagoon
Rio Angulito
Rio Negro

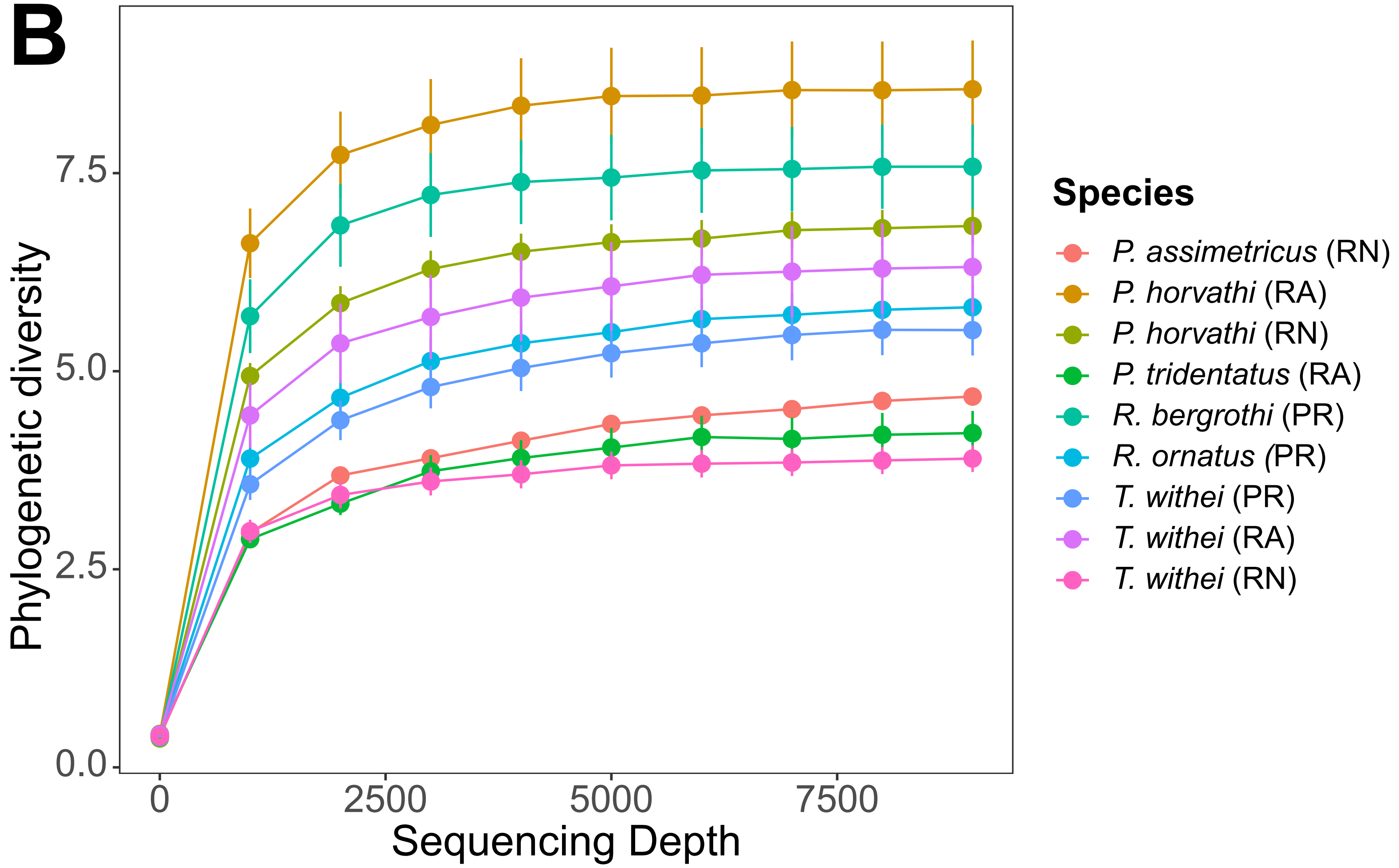

B
Phylogenetic diversity
7.5
5.0
2.5
0.0
0
2500
5000
7500
Sequencing Depth
Species
P. assimetricus (RN)
P. horvathi (RA)
P. horvathi (RN)
P. tridentatus (RA)
R. bergrothi (PR)
R. ornatus (PR)
T. withei (PR)
T. withei (RA)
T. withei (RN)
